# Long Noncoding RNA CBR3-AS1 Promotes Stem-like Properties and Oxaliplatin Resistance of Colorectal Cancer by Sponging miR-145-5p

**DOI:** 10.1155/2022/2260211

**Published:** 2022-04-12

**Authors:** Liangbao Xie, Guangfei Cui, Tao Li

**Affiliations:** Department of Gastrointestinal Hepatobiliary Surgery, The First People's Hospital of Shangqiu, Shangqiu 476400, China

## Abstract

Accumulating articles indicate that long noncoding RNAs (lncRNAs) serve as essential regulators in a plethora of human cancers. In this study, we analyzed the expression profile and functional role of lncRNA CBR3-AS1 in colorectal cancer (CRC). High-expression levels of CBR3-AS1 were found in CRC tissues and cell lines. Upregulated CBR3-AS1 was closely associated with poor prognosis and adverse clinicopathological features of CRC patients. Artificial knockdown of CBR3-AS1 markedly suppressed the proliferation, migration, invasion, and stem-like properties but promoted the apoptosis of CRC cells. Moreover, we observed that CBR3-AS1 could directly bind to miR-145-5p and negatively regulated its expression in CRC. Further experiments also demonstrated that inhibition of miR-145-5p reverted the effects of CBR3-AS1 knockdown on CRC cells. In addition, compared with the parental cells, CBR3-AS1 expression was strikingly increased in oxaliplatin- (OXA-) resistant CRC cells, and the OXA resistance was notably diminished by CBR3-AS1 knockdown. To conclude, our study suggested that CBR3-AS1 serves an oncogenic role in CRC and may be exploited as a novel therapeutic target for CRC patients.

## 1. Introduction

Colorectal cancer (CRC) is the third most prevailing cancer and a main cause of cancer-related death around the world [1]. Due to great advancements in the diagnostic and therapeutic methods, the survival rate of CRC patients has increased; however, due to the high frequency of recurrence and metastasis, the long-term prognosis of patients with advanced CRC remains unfavorable [2]. The occurrence and development of CRC is a complex process, and identification of novel therapeutic targets against this fatal malignancy is still urgently needed.

There is a large amount of noncoding RNA in the human genome. Long noncoding RNAs (lncRNAs), one group of transcripts with more than 200 nucleotides and deficiency of protein-coding potential, have been identified as critical determinants in many physiological and pathological processes, especially in the malignant progression of cancer [3, 4]. For example, CBR3-AS1, located on human chromosome 21q22.12, is a recently discovered cancer-related lncRNA. Zhang et al. reported that CBR3-AS1 predicts unfavorable prognosis and promotes tumorigenesis in osteosarcoma [5]. Besides, CBR3-AS1 functions as an oncogene and regulates radiosensitivity in non-small-cell lung cancer [6]. The aberrant expressions of lncRNAs are also tightly correlated to CRC oncogenesis [7], and in this study, we aimed to detect the expression of CBR3-AS1 in CRC and investigated its functional effects on CRC progression.

## 2. Materials and Methods

### 2.1. Patients and Tissue Samples

Primary CRC tissues and paired adjacent nontumor mucosa tissues were obtained from 133 patients who underwent surgical resection at hospital. None of these patients underwent chemotherapy or radiotherapy prior to surgery. After collection, all tissue samples were immediately snap-frozen in liquid nitrogen and stored at −80°C. This study was approved by the Ethics Committee of The First People's Hospital of Shangqiu. Written informed consent was obtained from all subjects in accordance with the Declaration of Helsinki.

### 2.2. Cell Culture and Treatments

CRC cell lines (HCT116, HT29, SW620, and SW480) and human normal colon epithelial cells (FHC), purchased from American Type Culture Collection (ATCC; Manassas, VA, USA), were cultured in Dulbecco's modified Eagle's medium (DMEM; Thermo Fisher Scientific, Inc., Waltham, MA, USA) containing 10% fetal bovine serum (FBS; HyClone, Logan, UT, USA) and 1% penicillin/streptomycin at 37°C in a humidified incubator with 5% CO_2_.

Oxaliplatin (OXA) was purchased from Meilun Biological Co., Ltd. (Dalian, China). OXA-resistant CRC cell lines (HCT116/OR and SW480/OR cells) were established by exposure to incremental doses of OXA (up to 2 *μ*M) for 3 months in our laboratory. OXA was removed before the experiments were performed.

The specific small interference RNA (siRNA) targeting CBR3-AS1 (si-CBR3-AS1), siRNA negative control (si-NC), miR-145-5p mimics, miR-145-5p inhibitor, miRNA mimics, and inhibitor negative control were synthesized by Guangzhou RiboBio Co., Ltd. (Guangzhou, China). These molecular products were transfected into cells using Lipofectamine 2000 (Invitrogen, Carlsbad, CA, USA). 48 h posttransfection, the transfection efficiency was analyzed by RT-qPCR analysis.

### 2.3. RT-qPCR Analysis

Total RNA was extracted using TRIzol reagent (Invitrogen) and quantified using a NanoDrop spectrophotometer (Thermo Fisher Scientific, Inc.). 200 ng RNA was reverse transcribed into cDNA using the PrimeScript RT reagent Kit (TaKaRa, Dalian, China), and PCR amplifications were then carried out using a SYBR Green PCR Kit (TaKaRa) on a 7500HT Real-Time PCR System (Applied Biosystems, Foster City, CA, USA). Data were analyzed using 2^−*ΔΔ*Ct^ method [8]. GAPDH or U6 was employed as an internal control.

### 2.4. Subcellular Fraction Location

Cytosolic and nuclear fractions of cells were isolated using the NE-PER™ Nuclear and Cytoplasmic Extraction Reagents Kit (Thermo Fisher Scientific, Inc.). Then, the expression levels of CBR3-AS1, U6, and GAPDH in the cytoplasm and nucleus were detected by RT-qPCR analysis.

### 2.5. MTT Assay

Cells (4,000 cells/well) were seeded in 96-well plates, and 20 *μ*l MTT solution (5 mg/l; Sigma-Aldrich, St. Louis, MO, USA) was added to each well. Following incubation for additional 4 h, 150 *μ*l DMSO (Sigma-Aldrich) was added to dissolve the formazan crystals. Then, the absorbance of each well at 570 nm was read on a microplate reader (Multiskan EX, Lab systems, Helsinki, Finland).

### 2.6. Cell Apoptosis Analysis

Cells were resuspended by 500 *μ*l 1× binding buffer and then double-stained with 5 *μ*l Annexin V-FITC and 5 *μ*l PI using an Annexin-V-PI Apoptosis Detection kit (BD Biosciences, Franklin Lakes, NJ, USA). Then, the samples were analyzed using a flow cytometry (FACScan; BD Biosciences) equipped with CellQuest software.

### 2.7. Transwell Assay

Cells in serum-free medium were put into the uncoated or Matrigel-coated upper chamber of Transwell plates (8 *μ*m pore size; Corning Inc., Corning, NY, USA), while 500 *μ*l medium containing 10% FBS as the attractant was loaded to the lower chamber. After 24 h, the cells on the lower membrane surface were fixed with 75% methanol and subsequently stained with 0.1% crystal violet. Then, the stained cells were captured using a microscope (Olympus, Tokyo, Japan).

### 2.8. Mammosphere Formation Assay

Single cells prepared by 0.25% trypsin-EDTA were seeded at a density of 2,000 cells/well in six-well ultralow attachment plates (Corning Inc.) and then cultured with serum-free DMEM/F12 medium (Thermo Fisher Scientific, Inc.) containing 20 ng/ml human EGF, 1% B27 and 20 ng/ml fibroblast growth factor for 7 days. The number of mammospheres (>20 *μ*m) was counted under an inverted microscope (Olympus).

### 2.9. Western Blot Analysis

Total protein was extracted using RIPA protein extraction reagent (Beyotime, Shanghai, China). The lysates were separated by SDS-polyacrylamide gel electrophoresis in 10% or 12% resolving gel and transferred to polyvinylidene difluoride membranes (Millipore, Bedford, MA, USA). The membranes were blocked with 5% skim milk powder for 2 h at room temperature, followed by incubation with the specific primary antibodies at 4°C overnight. The membranes were then hatched with HRP-conjugated secondary antibody at room temperature for 1 h. The immunoreactive bands were visualized with the enhanced chemiluminescence reagents (Bio-Rad Laboratories, Hercules, CA, USA). GAPDH served as the loading control.

### 2.10. Dual-Luciferase Reporter Assay

The fragment of CBR3-AS1 containing the predicted miR-145-5p-binding sites was cloned into the psiCHECK-2 luciferase reporter vector (Promega, Madison, WI, USA), and mutations in the binding sites were achieved using a Mut Express II Fast Mutagenesis kit (Vazyme, Piscataway, NJ, USA). Cells were cotransfected with the luciferase constructs and miR-145-5p mimics or mimics negative control using Lipofectamine 2000. After 48 h, the luciferase activities were measured using the Dual-Luciferase Reporter Assay System (Promega).

### 2.11. RNA Immunoprecipitation (RIP) Assay

RIP assay was performed using the EZ-Magna RIP™ RNA-Binding Protein Immunoprecipitation Kit (Millipore). The lysates of cells transfected with miR-145-5p mimics or mimics negative control was incubated with magnetic beads conjugated with anti-Ago2 or IgG antibody. The immunoprecipitated RNA was purified and subjected to RT-qPCR analysis.

### 2.12. Statistical Analysis

All statistical analyses were performed using GraphPad Prism 6.0 software (GraphPad Software, Inc., La Jolla, CA, USA) and SPSS 18.0 software (SPSS Inc., Chicago, IL, USA). The differences between groups were undertaken using Student's *t*-test, Chi-square test or one-way ANOVA followed by Tukey's test. Survival curves were generated by Kaplan-Meier analysis and compared using log-rank test. All *P* values< 0.05 were considered to indicate statistical significance.

## 3. Results

### 3.1. CBR3-AS1 Is Overexpressed in CRC

**Figure 1:**
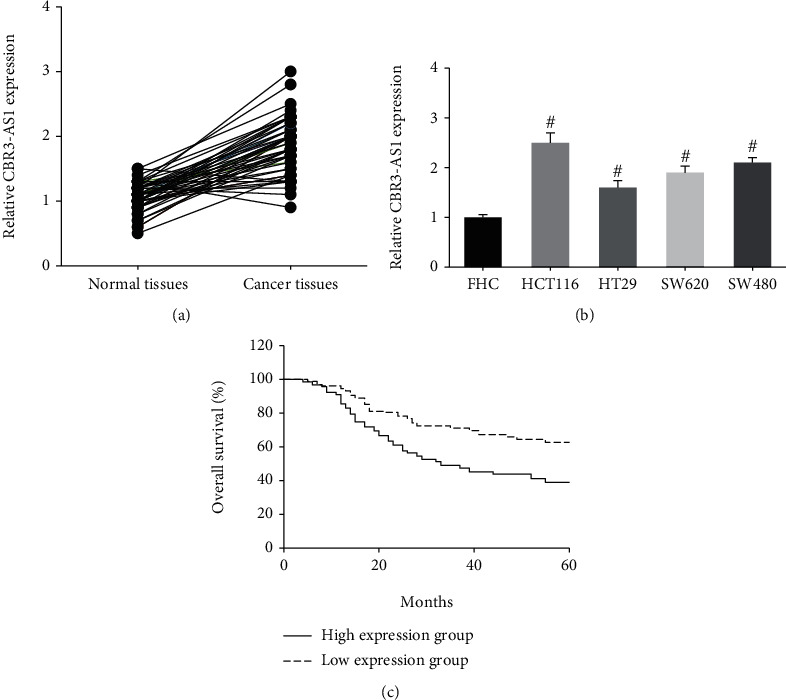
CBR3-AS1 is overexpressed in CRC. (a) RT-qPCR analysis of CBR3-AS1 expression levels in CRC tissues and adjacent normal tissues. (b) RT-qPCR analysis of CBR3-AS1 expression levels in CRC cell lines and FHC cells. (c) Kaplan-Meier analysis of correlation between CBR3-AS1 expression and overall survival of CRC patients. ^#^*P* < 0.05 vs. FHC cells.

As indicated by RT-qPCR analysis, CBR3-AS1 was significantly upregulated in CRC tissues, compared with their corresponding normal tissues (Figure 1(a)). Besides, the expression levels of CBR3-AS1 were markedly increased in CRC cell lines (HCT116, HT29, SW620, and SW480), compared to normal FHC cells (Figure 1(b)). HCT116 and SW480 cells, with the highest expression levels of CBR3-AS1, were selected for further investigation.

We then analyzed the correlation between CBR3-AS1 expression and the clinicopathological parameters of 133 CRC patients. They were allocated into the low-expression group (*N* = 70) and high-expression group (*N* = 63), according to the median value of CBR3-AS1 expression.  Table 1 showed that high CBR3-AS1 expression was closely related to larger tumor size (*P* = 0.034), distant metastasis (*P* = 0.037), and advanced TNM stage (*P* = 0.033) in CRC patients. Moreover, survival analysis showed that CRC patients with high CBR3-AS1 expression had shorter overall survival compared to those with low CBR3-AS1 expression (*P* = 0.006; Figure 1(c)).

### 3.2. CBR3-AS1 Knockdown Inhibits the Malignant Behaviors of CRC Cells

**Figure 2:**
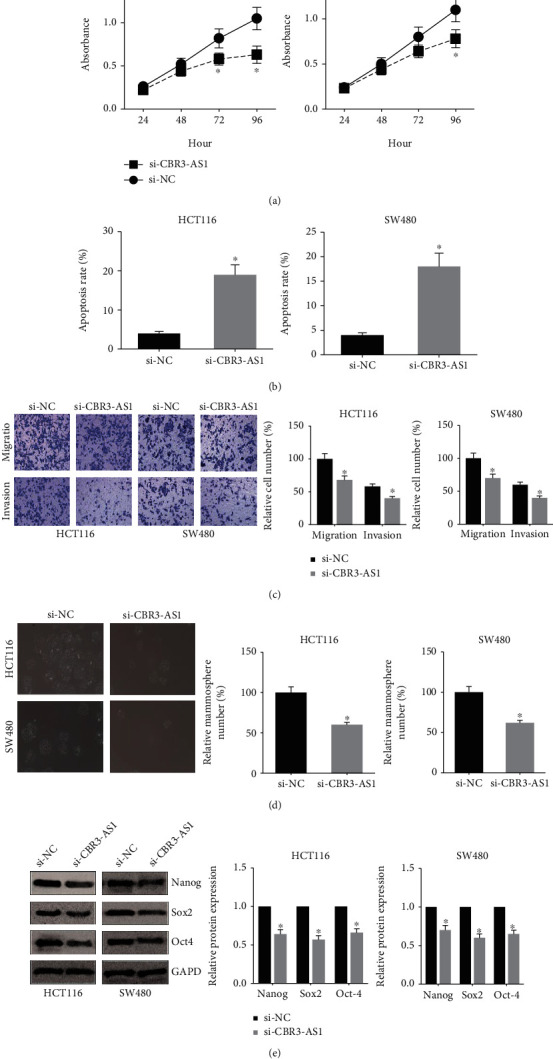
CBR3-AS1 knockdown inhibits the malignant behaviors of CRC cells. (a) MTT assay showed the proliferation of CRC cells after transfection. (b) Flow cytometry analysis showed the apoptosis of CRC cells after transfection. (c) Transwell assay showed the migration and invasion of CRC cells after transfection. (d) Mammosphere formation assay showed the number of CRC cell mammospheres after transfection. (e) Western blot analysis showed the expression levels of CSC markers in CRC cells after transfection. ^∗^*P* < 0.05 vs. si-NC-transfected cells.

To further investigate the biological function of CBR3-AS1 in CRC, HCT116, and SW480 cells were transfected with si-CBR3-AS1. 48 h after transfection, we found that CBR3-AS1 expression was markedly decreased in both cell lines (data not shown). MTT assay indicated that, after knockdown of CBR3-AS1, the proliferation of HCT116 and SW480 cells was remarkably suppressed (Figure 2(a)). In addition, as shown in Figure 2(b), CBR3-AS1 knockdown triggered the elevation of apoptosis rate in HCT116 and SW480 cells.

Moreover, Transwell assay showed that the migration and invasion capacities of HCT116 and SW480 cells were remarkably suppressed when CBR3-AS1 was silenced (Figure 2(c)). We then cultured HCT116 and SW480 cells in serum-free sphere formation medium, and we found that CBR3-AS1-silenced cells exhibited significantly fewer mammospheres (Figure 2(d)). The expression levels of CSC markers were detected by western blot analysis. As demonstrated in Figure 2(e), CBR3-AS1 knockdown markedly decreased the expression levels of Nanog, Sox2, and Oct4 in HCT116 and SW480 cells.

### 3.3. CBR3-AS1 Directly Binds to miR-145-5p and Inhibits Its Expression in CRC

**Figure 3:**
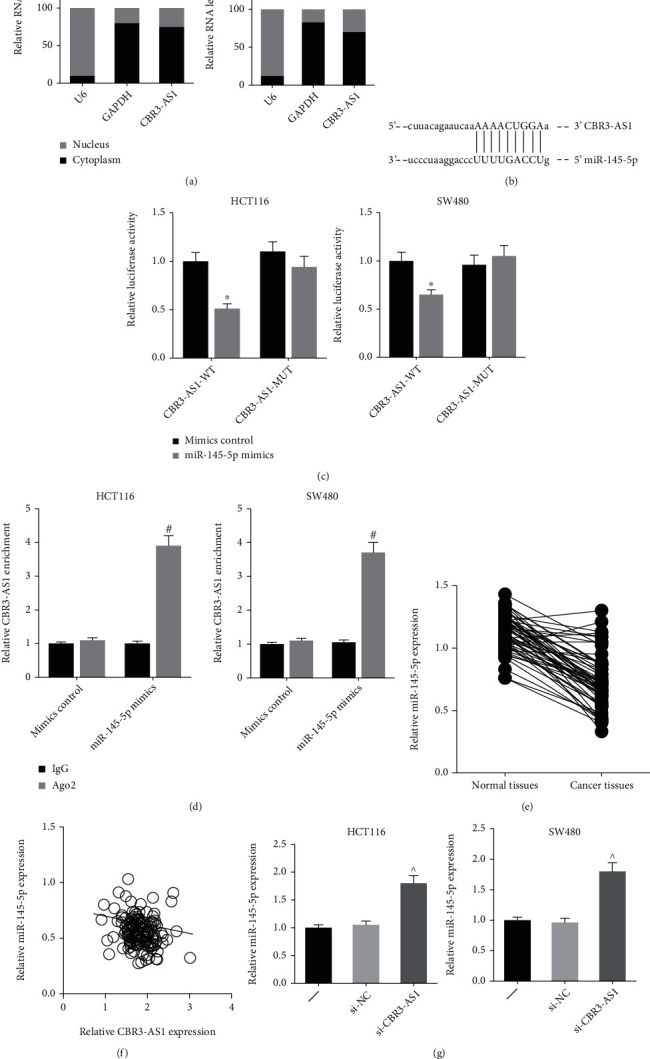
CBR3-AS1 directly binds to miR-145-5p and inhibits its expression in CRC. (a) Cell fractionation analysis showed the subcellular distribution of CBR3-AS1 in CRC cells. (b) The predicted binding site of miR-145-5p within CBR3-AS1 fragment. (c) Dual-luciferase reporter assay validated the binding relation between CBR3-AS1 and miR-145-5p in CRC cells. (d) RIP assay showed the enrichment of CBR3-AS1 in CRC cells after transfection. (e) RT-qPCR analysis of miR-145-5p expression levels in CRC tissues and adjacent normal tissues. (f) An inverse expression correlation between CBR3-AS1 and miR-195-5p in CRC tissues. (g) RT-qPCR analysis of miR-145-5p expression levels in CRC cells after transfection. ^∗^*P* < 0.05 vs. mimics control-transfected cells; ^#^*P* < 0.05 vs. IgG antibody; ^*P* < 0.05 vs. si-NC-transfected cells.

Given that CBR3-AS1 is mainly located in the cytoplasm of HCT116 and SW480 cells (Figure 3(a)), we therefore hypothesized that it may serve as miRNA sponge in CRC. Through the starBase database (http://starbase.sysu.edu.cn/index.php), miR-145-5p was predicted to directly bind to CBR3-AS1, with the binding region shown in Figure 3(b). Dual-luciferase reporter assay was further carried out to verify the prediction. As demonstrated in Figure 3(c), cotransfection with miR-145-5p mimics notably reduced the luciferase activity of CBR3-AS1-WT in HCT116 and SW480 cells, rather than CBR3-AS1-MUT. Through RIP assay, we also observed that CBR3-AS1 was strikingly enriched by Ago2 antibody in HCT116 and SW480 cells transfected with miR-145-5p mimics (Figure 3(d)).

Moreover, Figure 3(e) indicated that miR-145-5p was significantly downregulated in CRC tissues, and a negative correlation was further identified between the expression levels of CBR3-AS1 and miR-145-5p (Figure 3(f)). The expression of miR-145-5p was notably increased by si-CBR3-AS1 in HCT116 and SW480 cells (Figure 3(g)).

### 3.4. miR-145-5p Inhibition Blocks the Effects of CBR3-AS1 Knockdown in CRC Cells

**Figure 4:**
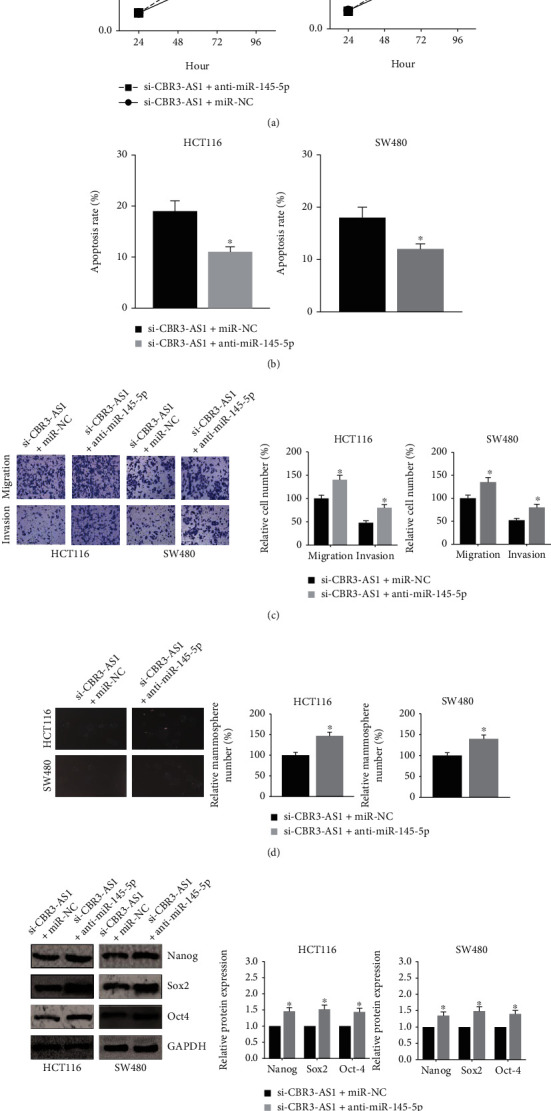
miR-145-5p inhibition blocks the effects of CBR3-AS1 knockdown in CRC cells. (a) MTT assay showed the proliferation of CRC cells after transfection. (b) Flow cytometry analysis showed the apoptosis of CRC cells after transfection. (c) Transwell assay showed the migration and invasion of CRC cells after transfection. (d) Mammosphere formation assay showed the number of CRC cell mammospheres after transfection. (e) Western blot analysis showed the expression levels of CSC markers in CRC cells after transfection. ^∗^*P* < 0.05 vs. NC inhibitor-transfected cells.

MTT assay further indicated that cotransfection with miR-145-5p inhibitor markedly rescued the impaired proliferation of HCT116 and SW480 cells after CBR3-AS1 knockdown (Figure 4(a)), and the increased apoptosis rates of these cells were also diminished by miR-145-5p inhibition (Figure 4(b)). Moreover, the inhibitory effects of si-CBR3-AS1 on the migration and invasion of HCT116 and SW480 cells were markedly blocked by miR-145-5p inhibition (Figure 4(c)). We further noticed that miR-145-5p inhibition increased the number of cell mammospheres (Figure 4(d)), accompanied by the increased expression levels of Nanog, Sox2, and Oct4 (Figure 4(e)).

### 3.5. CBR3-AS1 Knockdown Blocks OXA Resistance in CRC Cells

**Figure 5:**
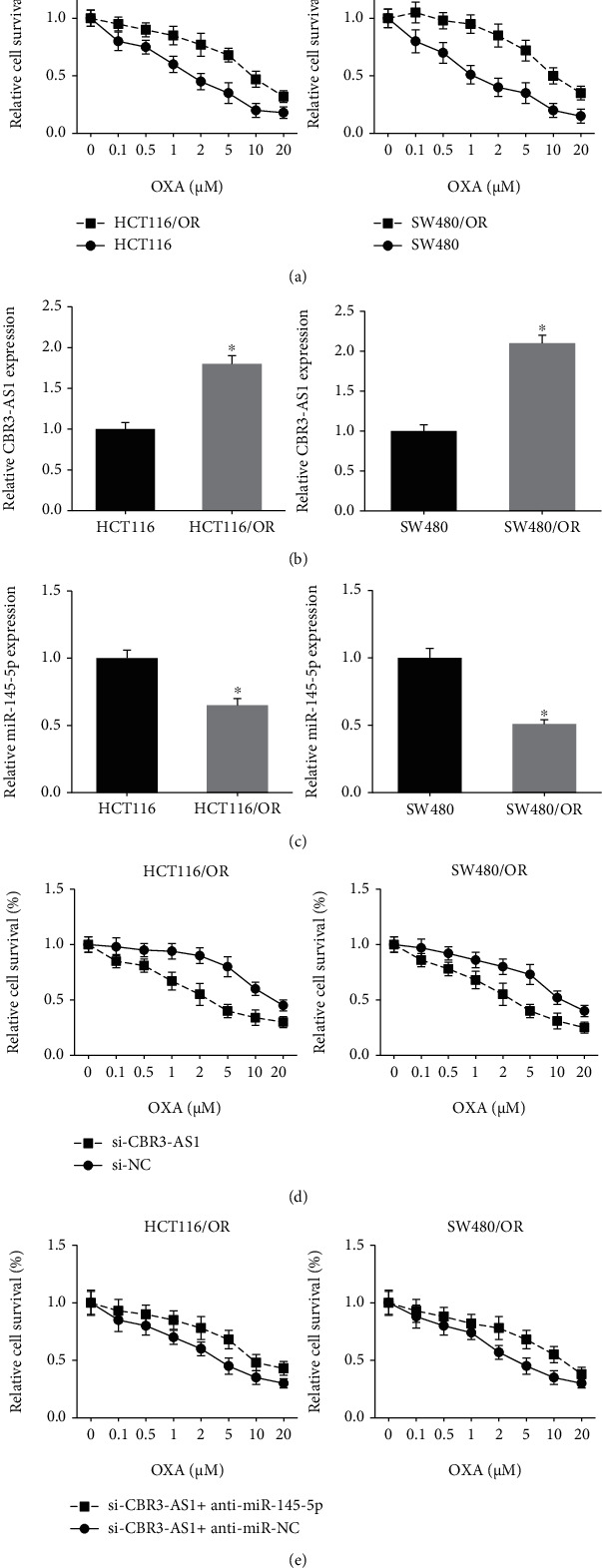
CBR3-AS1 knockdown blocks OXA resistance in CRC cells. (a) MTT assay showed the chemosensitivity of OXA-resistant CRC cells and parental cells to OXA. (b) RT-qPCR analysis of CBR3-AS1 expression levels in OXA-resistant CRC cells and parental cells. (c) RT-qPCR analysis of miR-145-5p expression levels in OXA-resistant CRC cells and parental cells. (d, e) MTT assay showed the chemosensitivity of OXA-resistant CRC cells to OXA after transfection. ^∗^*P* < 0.05 vs. parental cells.

We then established OXA-resistant CRC cell lines, and MTT assay confirmed that, compared with the parental cells, HCT116/OR and SW480/OR cells showed more resistance to OXA (Figure 5(a)). The expression levels of CBR3-AS1 were strikingly increased, while miR-145-5p was downregulated in HCT116/OR and SW480/OR cells (Figures 5(b) and 5(c)). Furthermore, as shown in Figures 5(d) and 5(e), CBR3-AS1 knockdown significantly enhanced the chemosensitivity of HCT116/OR and SW480/OR cells to OXA, but this effect was notably diminished by miR-145-5p inhibition.

## 4. Discussion

As a heterogeneous multifactorial disease, CRC continues to have a significant impact on global public health [9]. Overwhelming evidence has demonstrated that numerous lncRNAs are closely associated with CRC progression and development [10]. They have various expression patterns in CRC samples and can serve as oncogenes or tumor suppressors. Targeting lncRNAs and elucidating the underlying mechanisms may improve the efficacies of diagnostic and therapeutic methods for CRC.

In this study, CBR3-AS1 was found to be remarkably upregulated in CRC tissues and cell lines, and its high expression was closely correlated with poor prognosis and adverse clinicopathological features of CRC patients. By a series of loss-of-function assays, we further observed that CBR3-AS1 knockdown yielded significant inhibitory effects on the malignant properties of CRC cells, revealing its function as an oncogene. OXA is a first-line chemotherapeutic drug for CRC patients, but chemoresistance remains a major challenge [11]. Cancer stem cells (CSCs) play a critical role in the recurrence and chemoresistance of CRC, and targeting self-renewal of CSCs may represent a new paradigm in CRC therapy [12, 13]. In this study, we also confirmed that CBR3-AS1 knockdown suppressed the stem-like properties and OXA resistance of CRC cells.

As a class of short (~22 nt) noncoding RNAs, microRNAs (miRNAs) also significantly contribute to CRC progression and development [14]. Up to now, accumulating articles indicated that lncRNAs located in the cytoplasm always function as molecular sponges to compete miRNAs in a sequence-specific manner [15]. miR-145-5p was previously reported to be a tumor suppressor in most types of cancers, including CRC [16, 17], and in our study, by bioinformatics analysis and experimental verification, we confirmed that CBR3-AS1 could directly bind to miR-145-5p and suppressing its expression and function in CRC. Compared with the parental cells, miR-145-5p expression was also decreased in OXA-resistant CRC cells. Furthermore, the results of rescue experiments revealed that miR-145-5p inhibition blocked the effects of CBR3-AS1 knockdown in CRC cells.

In conclusion, the present study provided convincing evidence that CBR3-AS1 promotes stem-like properties and OXA resistance of CRC cells partly by sponging miR-145-5p. CBR3-AS1 could be considered a potential therapeutic target for CRC patients in the future.

## Figures and Tables

**Table 1:** The relationship between clinicopathological characteristics and CBR3-AS1 expression in 133 CRC patients.

Characteristics	Total number (*N* = 133)	CBR3-AS1 expression		*P* value
		Low (*N* = 70)	High (*N* = 63)	
Age (years)				0.236
<60	47	28	19	
≥60	86	42	44	
Gender				0.455
Male	80	40	40	
Female	53	30	23	
Tumor size (cm)				0.034
<5	74	45	29	
≥5	59	25	34	
Tumor differentiation				0.176
Well+moderate	100	56	44	
Poor	33	14	19	
Lymph node metastasis				0.215
No	56	33	23	
Yes	77	37	40	
Distant metastasis				0.037
No	84	50	34	
Yes	49	20	29	
TNM stage				0.033
I-II	72	44	28	
III-IV	61	26	35	

## Data Availability

The data used to support the findings of this study are available from the corresponding author upon request.
